# Mesenchymal Stem Cell-Derived Exosomes as a Neuroregeneration Treatment for Alzheimer’s Disease

**DOI:** 10.3390/biomedicines12092113

**Published:** 2024-09-17

**Authors:** Siddharth Shah, Hadeel M. Mansour, Tania M. Aguilar, Brandon Lucke-Wold

**Affiliations:** 1 Department of Neurosurgery, University of Florida, Gainesville, FL 32608, USAbrandon.lucke-wold@neurosurgery.ufl.edu (B.L.-W.); 2 College of Medicine, University of Illinois at Chicago, Chicago, IL 60612, USA

**Keywords:** Alzheimer’s disease, mesenchymal stem cells, drug discovery and development, neurosurgery, neurology, novel therapies

## Abstract

Background: Alzheimer’s disease (AD) is the most prevalent kind of dementia and is a long-term degenerative disease. Pathologically, it is defined by the development of extracellular amyloid-β plaques and intracellular neurofibrillary tangles made up of hyperphosphorylated tau protein. This causes neuronal death, particularly in the hippocampus and cortex. Mesenchymal stem cell (MSC)-derived exosomes have been identified as possibly therapeutic and have promise for Alzheimer’s disease due to their regenerative characteristics. Methods: A systematic retrieval of information was performed on PubMed. A total of 60 articles were found in a search on mesenchymal stem cells, exosomes, and Alzheimer’s disease. A total of 16 ongoing clinical trials were searched and added from clinicaltrials.gov. We added 23 supporting articles to help provide information for certain sections. In total, we included 99 articles in this manuscript: 50 are review articles, 13 are preclinical studies, 16 are clinical studies, 16 are ongoing clinical trials, and 4 are observational studies. Appropriate studies were isolated, and important information from each of them was understood and entered into a database from which the information was used in this article. The clinical trials on mesenchymal stem cell exosomes for Alzheimer’s disease were searched on clinicaltrials.gov. Results: Several experimental investigations have shown that MSC-Exo improves cognitive impairment in rats. In this review paper, we summarized existing understanding regarding the molecular and cellular pathways behind MSC-Exo-based cognitive function restoration, with a focus on MSC-Exo’s therapeutic potential in the treatment of Alzheimer’s disease. Conclusion: AD is a significant health issue in our culture and is linked to several important neuropathological characteristics. Exosomes generated from stem cells, such as mesenchymal stem cells (MSCs) or neural stem cells (NSCs), have been examined more and more in a variety of AD models, indicating that they may be viable therapeutic agents for the treatment of diverse disorders. Exosome yields may be increased, and their therapeutic efficacy can be improved using a range of tailored techniques and culture conditions. It is necessary to provide standardized guidelines for exosome manufacture to carry out excellent preclinical and clinical research.

## 1. Introduction

Alzheimer’s disease (AD) is the most common type of dementia and is a chronic progressive disease [1]. With advanced age being the strongest risk factor, AD affects one in nine people aged 65 or older, and over 6 million people currently suffer from AD in the USA alone [2]. The term “Alzheimer’s disease” (AD) dementia describes a specific neuropathology together with an age-related start and progression of cognitive and functional impairment. Alois Alzheimer initially wrote about it in 1906, describing a case he had seen in 1901. In addition to criteria designed for modern clinical diagnosis, criteria for using biomarkers to identify preclinical, or presymptomatic, phases of the illness have also been devised. Alzheimer defined the fundamental neuropathology, which later developed into a more precise neuropathologic diagnosis in the mid-1980s that acknowledges the concomitant neuropathologies that usually lead to clinical dementia [2,3]. Given the direct relation of age to the disease, AD has rapidly become a major public health problem worldwide, and the burden of AD is expected to increase with the ageing population with an estimation of 152 million by 2050 [2]. AD is now considered the fifth leading cause of death in the United States, with deaths doubling from 2000 to 2021 [3] and reported deaths increasing by 140% [2], underscoring the urgent need for early detection and new therapeutic interventions [4]. Pathologically, AD is characterized by the presence of extracellular amyloid-β plaques and intracellular neurofibrillary tangles made up of hyperphosphorylated tau protein, which results in neuronal death, especially in the hippocampus and cortex [5,6]. This neuronal degradation is expressed clinically as memory dysfunction, frontal lobe dysfunction, aphasia, and agnosia [7,8]. Risk factors include age, diabetes, cardiovascular disease, and genetic factors such as Down syndrome [9,10]. Additionally, studies have indicated that other factors such as physical inactivity and poor diet play a role in the development of AD [10,11]. Early in the disease, there are deficits in the capacity to encode and retain new memories. The latter phases are accompanied by gradual changes in cognition and behavior. Reduced synaptic strength, synaptic loss, and neurodegeneration are the results of altered amyloid precursor protein (APP) cleavage, the APP fragment beta-amyloid (Aβ), and hyperphosphorylated tau protein aggregation. Important elements of the illness process include concomitant diseases, alterations in metabolism, vascular function, and inflammation [11].

To this day, there is no cure for AD, and current therapeutic drugs, like Donepezil and Rivastigmine, only aim at alleviating the symptoms and temporarily improving cognitive function without arresting the progression of AD or repairing the massive neuronal loss that defines the disease [12,13,14]. Additionally, these drugs may have poor outcomes and side effects in elderly patients such as syncope and reduced cardiac output that further call for the importance of developing new therapeutic approaches [15]. The present research is investigating several new strategies, such as the regulation of neuroinflammation and the prevention of amyloid beta formation, but these procedures are still in the experimental phase [16]. 

Recent research has highlighted the potential of mesenchymal stem cell-derived exosomes (MSCs) as a novel therapeutic approach for AD. MSCs offer several advantages that make them promising candidates for AD treatment. First, they can release anti-inflammatory substances that decrease neuroinflammation—one of the key processes in the formation and progression of AD [17,18]. MSCs may help to arrest the progression of the disease by reducing the levels of chronic brain inflammation and transforming into neuron-like cells that may help in replacing the damaged or missing neurons, thereby helping in regaining the lost memory [19]. 

Recent research has also demonstrated that MSCs can also help in the prevention of the formation of amyloid beta plaques, which is one of the key factors of AD, and which, if prevented, could slow the progression of the disease and enhance the results of the treatment [20,21]. Moreover, the immunogenicity of MSCs is relatively low, which means that the body’s immune system will not reject the MSCs, and hence, MSC therapy is safe and effective for the treatment of AD [22,23].

Our study aims to explore and discuss the pathophysiology of AD and review recent findings on the therapeutic potential of MSC-derived exosomes, highlighting their role as a promising avenue for future treatment options. 

## 2. Pathophysiology of AD

Alzheimer’s disease (AD) is a neurodegenerative disease characterized by progressive memory loss and cognitive impairment. The pathophysiology of AD is primarily caused by three main hallmarks: the accumulation of amyloid beta (Aβ) plaques, also known as senile plaques; the presence of neurofibrillary tangles of hyperphosphorylated tau protein; and the marked neuronal degeneration. Aβ proteins are chemically “sticky” and generally build up to plaques that, if accumulated in the brain, can clump and block cell-to-cell signaling at synapses [1]. They also activate the brain’s immune system, which triggers inflammatory responses that further damage the disabled neuron cells [24,25]. Neurofibrillary tangles are tangles of the protein called tau, which is a microtubule-associated protein that stabilizes neuronal microtubules under normal physiological conditions; however, in AD, tau becomes phosphorylated, causing toxic aggregates that deposit within the neuron. 

These pathological changes are associated with the loss of cholinergic neurons, synaptic dysfunction, and glial activation, contributing to widespread atrophy of the hippocampus and subsequently the cerebral cortex. While the exact pathophysiology of AD as well as its treatment remains a mystery, there are two proposed hypotheses based on these pathologic abnormalities.

The Cholinergic Hypothesis: states that reduced levels of acetylcholine caused by neuronal loss play a crucial role in the development of Alzheimer’s disease. Acetylcholine is important for several physiological processes such as memory, attention, learning, and other critical cognitive functions hence why Beta-amyloid is believed to affect cholinergic function and impair acetylcholine release negatively [26]. 

The Amyloid Hypothesis: the widely accepted hypothesis suggests that Alzheimer’s Disease (AD) is caused by the accumulation of amyloid beta (Aβ) peptides, particularly Aβ42, which are derived from the amyloid precursor protein (APP) through the actions of β- and γ-secretase enzymes. Elevated levels of Aβ42 lead to the formation of toxic amyloid aggregates that damage neurons [26,27]. 

### Braak and Braak Staging

A staging method developed in the late 1980s by two scientists, T Heiko Braak and Eva Braak, divides the growth of neurofibrillary tangles into six phases. The National Institute on Aging and the Reagan Institute give generally acknowledged diagnostic criteria [28]. Neurofibrillary tangles show a better link with dementia severity in Alzheimer’s patients than amyloid plaques, yet amyloid remains a prominent hallmark of the illness. The pathogens of AD are sometimes compared to a “trigger and bullet” scenario [29]. Amyloid is thought to be the initial cause of the sickness. At the same time, tau, in the form of neurofibrillary tangles, works like a bullet that causes neurodegeneration and cognitive impairment. Accumulation of amyloid beta (Aβ) in cerebral blood vessels, known as cerebral amyloid angiopathy (CAA), can accelerate cognitive and memory impairment in Alzheimer’s disease patients [30]. 

## 3. Methods

The literature review was conducted in PubMed. The search terms used for this search were “alzheimers disease”, “AD”, “Mesenchymal Stem Cell”, “Exosomes”, “AD treatment”, “pathophysiology”, and “Advances in AD treatment”. This generated an initial search result of 244 articles. Articles were then screened for relevancy and then by abstract contents. These articles were filtered through to exclude keyword-mismatched articles, articles whose full texts were not available, articles not in the English language, and articles not related to AD. These came out to be 60 articles. A total of 16 ongoing clinical trials were searched and added from clinicaltrials.gov. We added 23 supporting articles to help provide information for certain sections. In total, we included 99 articles in this manuscript: 38 are review articles, 13 are preclinical studies, 28 are clinical studies, 16 are ongoing clinical trials, and 4 are observational studies. 

After this, relevant information from each article was entered into an Excel sheet with each section of this manuscript as a separate sheet to ease the process of data extraction for relevant sections. 

## 4. Neuroregeneration Therapy 

### 4.1. Stem Cells

Stem cells are a unique type of cells with the ability to proliferate, self-renew, and differentiate into various mature cell types. Stem cells have been used for decades, especially in Parkinson’s disease (PD), with significant success in numerous cell transplantation studies [23,31,32]. Therapeutic strategies involve direct cell replacement, secretion of neurotrophic and growth factors, and activation of endogenous neural precursor cells [33,34,35]. Regarding human embryonic stem cells’ clinical use, two difficulties exist: transplant rejection following implantation and ethical dilemmas surrounding the use of human embryos. Many teams have been attempting to use nuclear transfer to create hESCs from a patient’s somatic cells to get around these problems. Producing human nuclear transfer embryos is still a difficult technological task [36,37]. The ability of pluripotent stem cells (PSCs) to proliferate indefinitely has been a significant benefit, enabling the preparation of billions of diverse human cell types for transplantation. This characteristic has two drawbacks too, since tumors might develop if the cells continue to multiply even after transplantation [36]. There are three tumorigenic situations to think about. First, teratomas or tumors may develop as a result of improper patterning if undifferentiated and/or immature cells are kept in the final cell products that have been differentiated from human PSCs. Second, reprogramming factors may encourage carcinogenesis if they continue to be active in the iPS cells. Third, genetic alterations that have happened during PSC in vitro cultivation may be the source of tumorigenicity. Exosomes generated from mesenchymal stem cells (MSCs) have been shown to avert memory losses in an animal model of Alzheimer’s disease (AD). Except for the intended brain locations, other tissues have shown robust tracking of intravenously delivered exosomes. Here, we suggested targeting intravenously infused exosomes produced from MSCs (MSC-Exo) to the brain of transgenic APP/PS1 mice using the central nervous system-specific rabies virus glycoprotein (RVG) peptide. RVG and MSC-Exo were conjugated via a DOPE-NHS linker [35]. After intravenous administration, RVG-tagged MSC-Exo demonstrated enhanced targeting to the cortex and hippocampus. When RVG-conjugated MSC-Exo (MSC-RVG-Exo) was delivered to the group instead of MSC-Exo, there was a significant decrease in astrocyte activation, Aβ levels, and plaque formation. The Morris water maze test showed that brain-targeted exosomes produced from MSCs were superior to unmodified exosomes in improving cognitive performance in APP/PS1 mice. Moreover, while the intravenous injection of MSC-Exo decreased the production of pro-inflammatory mediators such as TNF-α, IL-β, and IL-6, the anti-inflammatory cytokines IL-10 and IL-13 did not exhibit significant alterations. On the other hand, MSC-RVG-Exo treatment markedly increased the levels of IL-10, IL-4, and IL-13 and markedly decreased the levels of TNF-α, IL-β, and IL-6 [35].

Human bone marrow-derived MSCs were cultivated in three-dimensional (3D) cell culture to assess the possible therapeutic benefits of MSC extracellular vesicles (EVs). Small EVs were then extracted by differential ultracentrifugation. For 4 months, non-transgenic (NT) or 5XFAD (familial Alzheimer’s disease mutation) mice received these tiny EVs intraperitoneally (IN) once every 4 days. After that, a range of behavioral tests were given to the mice to assess how their learning and memory had changed. Brain slices were then subjected to immunohistochemistry to determine the amounts of glial fibrillary acidic protein (GFAP) and amyloid beta (Aβ) [36]. The results showed that 5XFAD mice treated with hMSC-EV exhibited considerably improved behavior in cognitive tests when compared with 5XFAD mice treated with saline; however, there was no significant difference in behavior between EV-treated 5XFAD mice and NT animals. Furthermore, we observed a reduced Aβ plaque burden in the hippocampal regions of the mice treated with EV. Lastly, GFAP and Aβ plaque colocalization was reduced in the brains of mice treated with EV as opposed to saline [36].

There are multiple types of stem cells, including embryonic stem cells (ESCs), induced pluripotent stem cells (IPSCs), neural stem cells (NSCs), and mesenchymal stem cells (MSCs). In our study, we focus on evaluating the mesenchymal stem cell’s proposed role in treating Alzheimer’s disease. MSCs are adult multipotent cells that can be obtained from adult tissues such as bone marrow, skin, umbilical cord, adipose tissue, and spleen [36]. They can regenerate into different cell types, such as bone, cartilage, fat, lung, liver, and muscle [37]. They possess remarkable therapeutic potential, particularly in orthopedic applications. They also play roles in regenerative medicine and cancer treatment as anti-inflammatories, immunosuppressives, and vehicles for gene/protein therapy. Mesenchymal stem cells (MSCs) have been shown to promote the expression of anti-inflammatory factors like interleukin-10 and prostaglandin; however, it is important to explore the underlying mechanisms to determine if MSC transplantation directly influences inflammation or if the effects are due to tissue damage. Understanding this distinction is critical for optimizing MSC-based therapies. Further research is needed to clarify these mechanisms and their implications for treating neurodegenerative diseases [38]. In vitro, human MSCs can significantly increase the number of neurons in the hippocampus and induce neural precursor cells (NPCs) to differentiate into neurons via the Wnt signaling pathway. 

**Figure 1:**
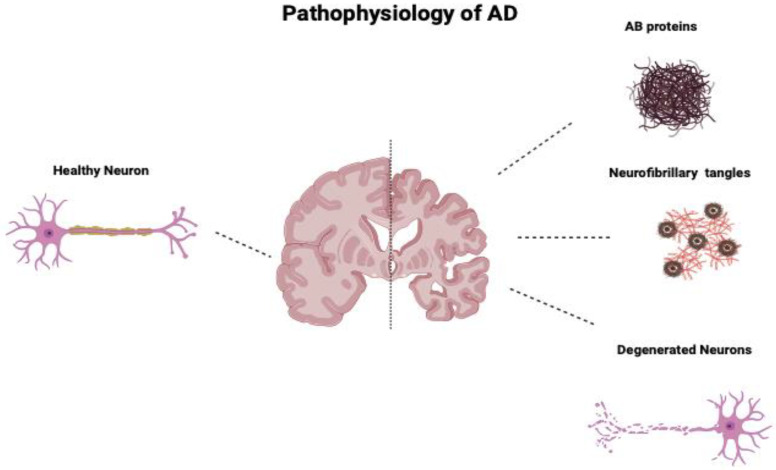
Comparison of neurons of a healthy cortex and those of a cortex diseased by Alzheimer’s. AD’s main pathologic changes are the accumulation of AB proteins, neurofibrillary tangles of tau protein, and loss and degeneration of neurons.

**Figure 2:**
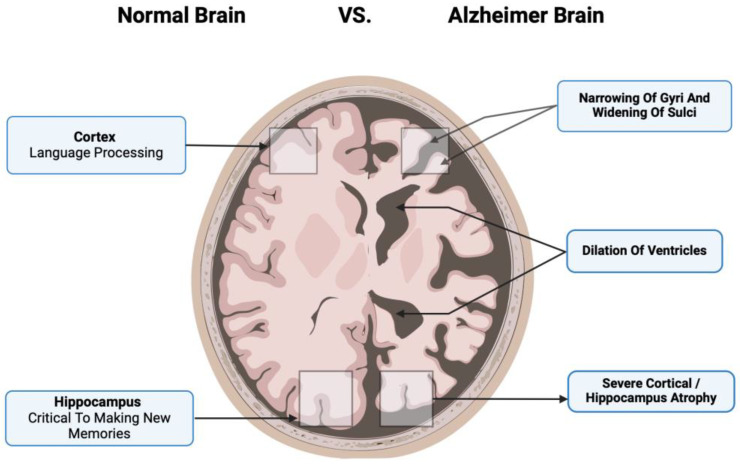
The main pathological markers in a diseased Alzheimer’s cortex include the narrowing of gyri and sulci, significant dilation of ventricles, and severe cortical atrophy that involves important brain functions such as language processing and making new memories.

Additionally, human MSCs can lower Aβ42 levels by stimulating autophagy both in vitro and in vivo [39]. Figure 1 compares the neurons of a healthy cortex and those diseased by Alzheimer’s. Figure 2 depicts the main pathological markers in a diseased Alzheimer’s. 

### 4.2. Exosomes

#### 4.2.1. Isolation of Exosomes

Ultracentrifugation: A conventional technique frequently employed to separate exosomes generated from stem cells is ultracentrifugation. Researchers can process a huge amount of samples with this technology. Large debris is first removed with low centrifugal force; then crude exosomal fractions are pelleted with high centrifugal force. Exosomes that have been identified and deemed crude are utilized in research without additional purification or are refined using density gradient ultracentrifugation [40].

Size-Based Filtration: To exclude any further extracellular vesicles that are bigger than 150 nm or less than 50 nm, biofluid samples can be run via size-exclusion chromatography or certain pore-sized filters. Exosome enrichment is not possible with this technique. After these filtering stages, ultracentrifugation might be utilized if exosome enrichment is required [41].

Precipitation of Polymers: To collect the vesicles with an exosomal size range (30–150 nm) and decrease exosomal solubility, which permits exosomes to precipitate, biofluid samples are mixed with a polymer, such as polyethylene glycol (PEG), to use polymer precipitation techniques for exosome purification. With standard laboratory equipment, this method is possible, but it depends on the polymer net size [42].

Immunoaffinity: The ability to withstand the particular proteins (antigens) found on exosomal membranes serves as the foundation for this technique. Particular antibodies coupled to a carrier, such as agarose or magnetic beads, can be used to extract a particular subtype of exosomes with great purity [43]. This approach is extensively employed in many applications, including fundamental research and clinical investigations, such as illness diagnosis and prognosis, because it has no volume constraint and is easily carried out with ordinary laboratory instruments. However, the materials needed for this process are often pricey [44]. 

#### 4.2.2. Cell Culture

Mesenchymal stem cells (MSCs) are grown under carefully controlled conditions to ensure that they develop properly for research or therapeutic purposes. Usually, MSCs are cultured in a basic growth medium like Dulbecco’s Modified Eagle Medium (DMEM), which is often enriched with fetal bovine serum (FBS). Fetal bovine serum (FBS) is used to provide the nutrients and growth factors needed for the cells to grow well. To keep the cultures free from contamination, a mix of antibiotics and antifungal agents is added. These cells are usually maintained in an environment with controlled humidity and 5% CO_2_ at a temperature of 37 °C, which closely resembles their natural surroundings. In clinical applications, it is crucial to decrease or eliminate animal-derived components, so serum-free media are often used. The cells are then cultured until they reach a certain level of growth, which ensures that they stay healthy and maintain their ability to develop into different types of cells [45]. 

#### 4.2.3. Working Model Biogenesis, Secretion, and Uptake

To understand the process of exosome synthesis, secretion, and uptake, tremendous effort has been made. The early sorting endosomes (ESEs) are first formed by endocytosis of external components and cell surface proteins, together with the inward budding of the plasma membrane. Intraluminal vesicles (ILVs) are formed by the invagination of the limiting endosomal membrane during the maturation phase of endosomes [46]. Many molecular machinery components influence the production of ILVs, but the endosomal sorting complex needed for transport (ESCRT) machinery complex is the primary regulator of this process. About thirty proteins make up the ESCRT mechanism, which assembles into four complexes (ESCRT-0, ESCRT-I, ESCRT-II, and ESCRT-III) and related proteins (including Vps4, Alix, and Tsg101) that are involved in the production of ILVs [47]. The ESCRT-I/II/III complex induces membrane deformation, ESCRT-0 sequesters ubiquitinated cargo proteins, and the Vps4 complex facilitates vesicle scission and recycling of the ESCRT-III complex [48]. 

A different pathway of exosome biogenesis, including tetraspanins, ceramides, cholesterol, phosphatidic acids, and heat-shock proteins (HSPs), is produced apart from the processes of the ESCRT machinery [49]. RNA loading into exosomes via lipid mediation relies on cargo domains and self-organizing lipids. Then, cytoplasmic substances including RNA, proteins, and lipids are encased in the lumen and gathered inside the late endosome to form multivesicular bodies (MVBs) [50]. The Golgi complex and endoplasmic reticulum play a role in the process. Some MVBs are carried to lysosomes for disintegration by fusing with autophagosomes or not, while other MVBs fuse with the plasma membrane through the cytoskeletal and microtubule network of the cell, eventually releasing their vesicles into the extracellular environment as exosomes [51]. 

Ceramides are more abundant in secreted MVBs than in degradative MVBs. It has been suggested that the distinct outcomes experienced by MVBs might be connected to the coexistence of subpopulations inside cells. Exosomal markers include proteins including flotillin, Alix, TSG101, tetraspanins (CD9, CD63, and CD81), and the endosome pathway, which is involved in exosome creation and release [52]). Furthermore, ceramide and sphingomyelin, two components of the lipid raft, are highly concentrated in exosomes [53]. 

## 5. Exosomes as AD Biomarkers

Currently, biomarkers of AD pathology (Aβ1-42/1-40, T-Tau, and p-Tau), cognitive behavioral syndrome (CBS), and positron emission tomography (PET/CT) are the major methods used to diagnose AD. However, because AD has a latent onset, bioimaging (PET/CT) and CBS-based diagnosis are frequently delayed. Biomarkers for monitoring, particularly with CSF, are intrusive and cause harm to patients. Currently, there are no reliable techniques for diagnosing or predicting AD [54]. AD is diagnosed in the clinic using a variety of methods, such as bioimaging, biochemical analysis, and questionnaires. The results of bioimaging, such as PET or CT, might be influenced by other dementia disorders, and the procedure is expensive. Surveys are prone to subjectivity and are often influenced by the survey taker [54]. Neuron adhesion molecules and neurotransmitter receptors are two examples of the distinctive receptors found in nervous tissues present in exosomes generated from neurons. The mediating function of those receptors is essential for the interactions that exosomes have with target cells. They make it easier for exosomes to bind and be taken up selectively, which allows their “cargo” to be delivered to certain cellular targets [55]. 

These receptors’ presence on exosomes makes it easier to use them for diagnostic purposes in neurodevelopmental disorders (NDDS). Blood, urine, and saliva are just a few of the bodily fluids from which exosomes from AD patients may be separated. Therefore, the ease of collection and the non-invasiveness of exosomes, in addition to their stability following sample capture, further validate their usefulness in the field of AD and associated illnesses diagnoses. Ruihua Sun et al. showed that exosomes obtained from the blood of AD patients were reduced in size and number compared with those from healthy controls using transmission electron microscopy (TEM) and nanoparticle tracking analysis (NTA) [56]. 

Antonio Longobardi et al. discovered that AD patients’ blood had 40% fewer exosomes than that of healthy controls, which is in line with that conclusion [57]. Exosomes from AD patients, according to different research, were bigger than those from healthy controls. At the moment, there is insufficient evidence to substantiate the precise variations in exosome size between AD patients and healthy controls. Exosome size variation may be influenced by several variables, such as sample origins, methods of collection, and procedures of analysis. To confirm these variations, learn more about their significance in the pathophysiology of AD, and assess their potential diagnostic use, more research is required [58]. Exosome morphology may be one factor in the diagnosis of AD; however, standardizing the methods for extracting and examining exosomes is necessary. An essential part of exosomes is proteins. β-site APP cleaving enzyme 1 (BACE-1), soluble peptide APP beta (sAPPβ), soluble peptide APP alpha (sAPPα), γ-secretase, and Aβ1-42 were detected in exosomes obtained from AD patients [59]. These findings are strongly associated with the etiology and development of AD. 

Exosome lipids have the potential to be useful biomarkers for the diagnosis of AD. Su et al. discovered using semi-quantitative mass spectrometry that brain-derived exosomes from AD patients had considerably higher levels of lipids and plasmalogen glycerophosphoethanolamine (PE) molecules (p-36:2, p-38:4) on their membranes than those from the control groups [60]. Another type of “cargo” from exosomes, miRNAs, has drawn more attention because of their function in regulating gene expression. It was established that exosomes from AD patients have significantly different miRNAs compared with exosomes from healthy controls [61]. According to Liu et al., in exosomes from the serum of AD patients, 19 miRNAs (such as miR-15a-5p) were elevated while 5 other miRNAs (such as miR-15b-3p) were downregulated. Microarray analysis was used to examine the expression levels of miRNA in the CSF of AD patients [46,62]. Gamez-Valero et al. discovered that the expressions of miR-132-5p, miR-485-5p, and miR-125b-5p were up, while those of miR-16-2, miR-29c, and miR-331-5p were lower [63]. The “cargo” and amounts of biomarkers formed from exosomes changed, indicating their great potential for use in the diagnosis of AD. Exosomes obtained from diverse bodily fluids guarantee their accessibility and availability for diagnosis purposes. 

Moreover, exosomes obtained from neurons and blood exhibit superior credibility in comparison with CSF biomarkers or PET/CT. Exosome markers can be combined with AD biomarkers, such as amyloid peptides or tau, and this combination can potentially improve AD diagnosis. Exosomes, which are small extracellular vesicles released by cells, carry molecular contents like proteins, lipids, and RNA, reflecting the state of their cells of origin. In Alzheimer’s, exosomes derived from neurons can contain amyloid beta and tau proteins, which are key pathological markers of the disease. By analyzing these exosomal contents in bodily fluids like blood or cerebrospinal fluid, it is possible to detect AD-related changes with high sensitivity [63]. Combining exosome markers with traditional biomarkers enhances diagnostic accuracy by providing a more comprehensive profile of the disease, potentially allowing for earlier detection and better monitoring of disease progression. Table 1 summarizes the biomarkers in diagnosing AD as reviewed by Guo et al. [17]. 

Peripheral blood brain-derived exosomes have demonstrated significant promise as the perfect “liquid biopsy” for AD. Blood-derived exosomes are distinguished by very sensitive and specific low-invasive diagnostic techniques. A lumbar puncture, neuroimaging, cognitive testing, and symptoms all play a role in the clinical diagnosis of AD. Though their quantity is smaller than that of cerebrospinal fluid, brain-derived exosomes have the intriguing ability to cross the blood–brain barrier and enter peripheral blood circulation. Researchers use immunoprecipitation techniques to separate brain-derived exosomes from plasma in order to get around these restrictions. Additionally, several studies indicate that CSF cannot reliably differentiate AD patients from patients with other forms of dementia due to the overlapping levels of Aβ1-42, T-tau, and p-tau181 [55,56]. Interestingly, a multicenter investigation verifies the association between blood and CSF levels of AD-associated protein. According to earlier studies, neuron-derived exosomes (NDEs) have lysosomal and synaptic protein levels that can be used to predict the preclinical risk of dementia development from moderate cognitive impairment (MCI). Functionally specialized synaptic protein NDE levels may be a good indicator of how severe AD is developing. Additionally, there appears to be a correlation between the illness stage and the quantities of complement proteins in ADEs, which are exosomes generated from astrocytes. The cargo proteins found in plasma ADEs are much more than those seen in NDEs, suggesting a possible target for BACE-1 inhibitors. Large cohort studies are also required to evaluate the diagnostic usefulness of exosomes, as well as a consistent preparation and biomarker procedure [56,57,58].

## 6. Therapeutic Properties of Exosomes and Application in Alzheimer’s Disease

MSCs are preferred to other stem cells, such as iPSCs or NSCs, for Alzheimer’s disease treatment due to their strong immunomodulatory properties, which help reduce neuroinflammation, a key factor in AD progression. They secrete neurotrophic factors like BDNF and VEGF, supporting neuron survival, neurogenesis, and synaptic plasticity. Unlike iPSCs, MSCs have a lower risk of tumorigenicity, making them a safer option. They are also easier to source and expand from tissues like bone marrow and adipose tissue, with low immunogenicity allowing for allogeneic transplantation without extensive immunosuppression. Their established clinical safety, coupled with their therapeutic effects through paracrine signaling and extracellular vesicle release, makes MSCs particularly attractive for AD treatment compared with the more complex and risk-prone iPSCs and the more lineage-specific NSCs [21,72].

Mesenchymal stem cells (MSCs) have been presented as a potential treatment and have shown promise for AD for their regenerative properties, such as secretion of growth factors, anti-inflammatory proteins, membrane receptors, and microRNAs (miRNAs) that can block apoptosis, decrease neuronal loss, and stimulate neurogenesis, synaptogenesis, and angiogenesis [73]. Their anti-apoptotic and antioxidant qualities aid in preventing neuronal cell death. Furthermore, MSCs secrete growth factors that encourage neural progenitor cells to improve neurogenesis, such as glial cell line-derived neurotrophic factor (GDNF) and brain-derived neurotrophic factor (BDNF). MSCs produce neurotrophins, including VEGF, HGF, NGF, BDNF, and neurotrophin-3, after they migrate to injured brain regions and interact with brain cells [20,59,60,74]. These neurotrophins support neuritic formation and neurorestoration, which helps with neurological recovery.

Moreover, MSCs regulate the immune response by suppressing inflammatory microglia (M1) and activating anti-inflammatory microglia (M2), which helps in preventing tissue damage caused by chronic neuroinflammation. They also promote the accumulation of microglia around Aβ deposits to increase Aβ clearance and to stimulate autophagy that aids in the lysosomal removal of Aβ plaques. These actions contribute to the therapeutic potential of MSCs in neurodegenerative disease treatment [43,75]

Numerous studies reveal that soluble factors produced from MSCs can alter the neuroprotective characteristics of Alzheimer’s disease (AD) models. For instance, Kim et al. reported that MSCs derived from human umbilical cord blood have a neuroprotective effect against Aβ toxicity in vitro by secreting galectin-3. Moreover, transplanting these MSCs into mice with AD transgenics causes microglia to produce MME/neprilysin, which improves Aβ clearance through soluble ICAM-1 secretion [76].

The characteristics of exosomes change depending on the cargo content, which is influenced by the extracellular environment and physiological conditions of the cell source. Therefore, it is crucial to take the effectiveness of MSC-exos into account when the environment changes. As stem cells, MSCs have a tremendous capacity for adaptability. The contents and biological activity of released exosomes are influenced by pretreatment MSCs in vitro, according to a number of recent investigations. Exosomes derived from hypoxia-preconditioned MSCs (PC-MSCs) have been shown to improve the treatment outcome in mice with AD transgenics. A pretreatment group benefits mostly in enhanced learning and memory, reduced Aβ buildup, elevated production of synaptic proteins, and reduced inflammatory response [35]. It is interesting to note that, under standard growth conditions, MSC-EVs did not exhibit the previously reported therapeutic benefits. It is also important to remember that MSCs probably change the properties of exosomes in pathological circumstances. Conversely, several investigations demonstrated that, by delivering physiologically active components, MSC-EVs changed the cellular metabolic milieu. Thus, the therapeutic potential of MSC-exos in AD may be improved by ongoing modifications of MSC pretreatment techniques, at least somewhat.

**Figure 3:**
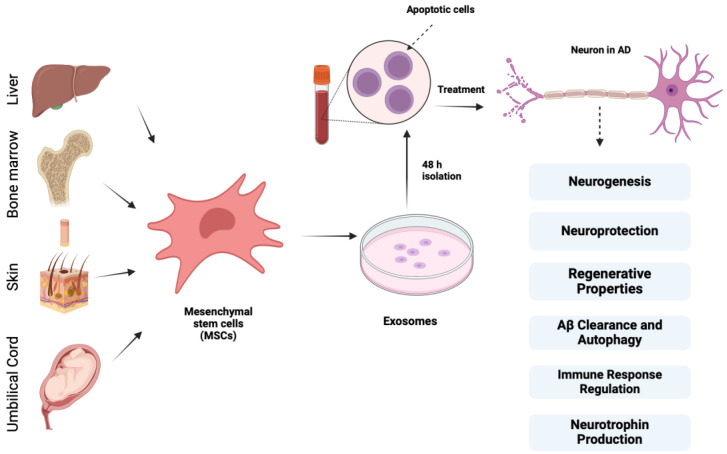
Different sources of mesenchymal stem cells and their properties of importance in AD treatment along with exosomes.

According to Lee et al., bone marrow-derived MSCs exert neuroprotective effects on AD models that are underpinned by cellular and molecular mechanisms, and CCL5, which is secreted from blood-derived MSCs, recruits alternative microglia to the AD brain, thereby reducing Aβ deposition and memory impairment through the production of IL-4 and MME [77,78]. These data indicate that MSC treatment in Aβ-treated cells remarkably boosts autolysosome formation and autolysosomal catabolic function, which contribute to enhanced neuronal survival. Figure 3 depicts different sources of mesenchymal stem cells and their properties of importance in AD.

MSCs derived from various sources, such as bone marrow, adipose tissue, umbilical cord blood skin, and liver, exhibit regenerative properties by secreting growth factors, anti-inflammatory proteins, membrane receptors, and miRNAs that block apoptosis, decrease neuronal loss, and stimulate neurogenesis, synaptogenesis, and angiogenesis. They provide neuroprotection through their anti-apoptotic and antioxidant effects and promote neurogenesis by releasing growth factors like GDNF and BDNF. Additionally, MSCs produce neurotrophins such as VEGF, HGF, NGF, BDNF, and neurotrophin-3, supporting neuritic formation and neurorestoration and regulating immune response by suppressing inflammatory microglia (M1) and activating anti-inflammatory microglia (M2), preventing tissue damage and enhancing Aβ clearance through microglial accumulation and autophagy stimulation.

## 7. Clinical Trials

Numerous studies have been carried out to determine the effectiveness of MSCs obtained from different origins in managing Alzheimer’s disease (AD). However, there are only a few of these trials that are officially completed and have published their results. Table 2 presents a list and summary of the clinical trials registered on clinicaltrials.gov. These trials are significant for the further development of MSCs as a therapeutic target for AD, showing both the achievements and the challenges in this novel therapeutic approach.

Several clinical trials have been conducted in the past years to evaluate the use of MSCs in AD, using different sources of stem cells, routes of administration, and study protocols. Some of the completed studies have given preliminary information on the safety and possibility of undertaking these therapies. The intravenous application of bone marrow-derived MSCs was investigated in a study conducted in the USA with 33 AD patients; the treatment was safe, but there was no significant improvement in the cognitive status of the patients [79]. Likewise, another trial in the USA included 21 patients who were treated with adipose tissue-derived MSCs through intravenous administration; however, the study’s small sample size limited the conclusions that could be drawn [80]. Another study conducted in South Korea used intracerebroventricular infusion of umbilical cord blood-derived MSCs (UCB-MSCs) in 45 patients and proved that this method of delivery is safe, but its effect on the progression of the disease is still ambiguous [81]. A similar trial in South Korea involved the intracerebral transplantation of UCB-MSCs in 9 patients, and while the study showed that the treatment was safe, the data to support its therapeutic use were weak [82]. Furthermore, there are active and future trials aiming at testing MSC therapies for AD employing various approaches. Another trial that is still recruiting patients in the USA plans to investigate the safety and the efficacy of intravenous delivery of bone marrow-derived MSCs in 40 patients [83]. Another ongoing trial is assessing the safety of intravenous administration of umbilical cord-derived MSCs in 6 patients, but the trial is not recruiting, nor are there any results at the moment [84]. Current and future trials include a trial in the USA recruiting 80 participants to assess the safety and anti-inflammatory effects of adipose tissue-derived MSCs when administered intravenously and a trial in South Korea to evaluate the efficacy of intracerebroventricular infusion of UCB-MSCs in 9 patients to explore the effectiveness of a targeted delivery system [85,86]. Certain trials may have indefinite statuses or may have been affected by a reason. For instance, two trials in China are evaluating the intravenous umbilical cord-derived MSCs and UCB-MSCs in 16 and 30 patients, respectively, but their status is not known [87,88]. Another trial from South Korea aims at examining the safety and efficacy of intracerebroventricular administration of UCB-MSCs in 9 patients, but the trial’s status is also unknown [89]. Moreover, a phase 1 trial in the USA aimed at assessing the intravenous injection of adipose tissue-derived MSCs in 24 patients, but it was terminated due to the COVID-19 pandemic [90]. Other researchers are using different and innovative strategies and different cell types. There is only one study conducted in the USA in which adipose tissue-derived MSCs were used in a single patient, which does not allow us to make any conclusions [91]. Currently, there is an ongoing trial in China that has 9 participants in which the researchers aim to evaluate the safety and efficacy of adipose tissue-derived MSCs delivered through nasal drip [92]. Additionally, there are other ongoing trials without the distinction of the MSC source: a trial in South Korea is testing MSC administration through the intravenous route, and a registered large-scale clinical trial in the USA plans to enroll 5000 patients to examine MSC therapy safety and efficacy [93,94].

## 8. Advantages and Challenges

Mesenchymal stem cells’ (MSCs) ability to differentiate into various cell types, including those involved in the production of bone, cartilage, and adipose tissue, makes them highly advantageous for use in neurodegenerative diseases. Some studies have shown their anti-tumorigenic effects, such as Clarke et al., who stated that breast cancer cells cultured in an MSC-conditioned medium exhibit significant migratory inhibition compared with cells cultured in a standard medium [95,96]. Similarly, Bruno et al. showed tumor cell growth inhibition by MSCs. A human hepatocellular carcinoma cell line (HepG2), a human ovarian cancer cell line (Skov-3), and Kaposi’s sarcoma cell lines co-cultured in the presence of BM-MSCs exhibited reduced in vitro growth [97,98]. MSCs can also be obtained using minimally invasive means, such as bone marrow, adipose tissue, and umbilical cord blood. They can affect the immune system function and reduce inflammation, which is very helpful for treating inflammatory and autoimmune diseases. Their therapeutic value is increased by the minimal risk of immunological rejection in transplant recipients.

While SCs have the potential to repair and regenerate damaged cells, the precise ways in which they might work are still not fully understood. Most studies show that a single transplantation of MSCs is safe and does not induce an immune response. However, repeated administration of MSCs may result in the production of alloantibodies. So far, there have been only a few clinical trials where SCs were transplanted into AD patients, and results from animal studies have not provided solid proof that these therapies are either safe or effective. Andrzejewska et al. also reported antibacterial activities and interactions of the MSC secretome with cancer cells [99]. Additionally, there are a lot of social, ethical, and regulatory issues that make research difficult and limit federal funding. In the USA, the FDA has only approved stem cells from cord blood, but many clinics are offering various unregulated treatments, often charging a lot of money. To make sure that these treatments are safe and effective, especially for complicated diseases like Alzheimer’s, it is really important to have ongoing patient monitoring and clearer regulatory guidelines.

## 9. Conclusions

MSC-Exos play a crucial role as a mediator in the information transfer between MSCs and recipient cells, such as microglia and neurons. Improvements in cognitive function are brought about by MSC-Exo-derived miRNAs, trophic factors, enzymes, immunomodulatory agents, and pro-angiogenic chemicals, which stimulate neurogenesis and inhibit inflammation-induced damage to hippocampus neurons. Crucially, MSC-Exos generated immunomodulation and neuroprotection that was either identical or superior to that of their parent MSCs in terms of immunomodulation. The effects of MSC-Exos are independent of the local tissue microenvironment. MSC-Exos are immunomodulatory and neuroprotective cells that do not change in response to various stimuli, unlike MSCs, which change in phenotype and function upon engraftment in different tissue microenvironments. This suggests that MSC-Exos may find clinical application in treating neurocognitive diseases. MSC-expos are a unique cell-free therapeutic agent that offers incomparable benefits over cell-based therapy, which is thought to be a potential substitute in the treatment of AD.

## Figures and Tables

**Table 1:** Summary of biomarkers in the diagnosis of AD and their source. (↑—Increased, ↓—Decreased).

Source	Sample	Biomarker Protein Change
Neural	Plasma	P-T181-tau, P-S396-tau, and Aβ1–42 ↑, NRGN, REST ↓ compared with CNC and stable MCI patients [64].
Neural	Plasma or serum	Total Tau, P-T181-tau, P-S396-tau, and Aβ1–42 ↑compared with controls [65]
Neural	Plasma	Cathepsin D, LAMP-1, ubiquitinylated proteins ↑, and HSP70 ↓ compared with controls and FTD [66]
Neuronal	Plasma or serum	Aβ42, T-tau, and P-T181-tau ↓ compared with a MCI and control group Intracerebroven [67]
Neuronal	Plasma	Synaptophysin, synaptopodin, synaptotagmin-2, and neurogranin ↓ compared with controls [68]
Neuronal	Plasma	NPTX2, NRXN2α, AMPA4, NLGN1 ↓ [69]
Astrocyte	Plasma	complement proteins, IL-6, TNF-α, IL-1β ↑; complement regulatory proteins (CD59, CD46, DAF), complement receptor type 1 ↓ compared with controls [70]
Astrocyte	Plasma	BACE-1, (s)APPβ ↑, GDNF ↓ compared with controls [71]

**Table 2:** Completed and ongoing clinical trials on the effects of mesenchymal stem cells (MSCs) on Alzheimer’s disease patients.

	Number	Therapy	Source	Status	Pathway	N=	Study Location
1	NCT02600130 [79]	Cells	Bone marrow	Completed	Intravenous	33	USA
2	NCT03117738 [80]	Cells	Adipose tissue	Completed	Intravenous	21	USA
3	NCT02054208 [81]	Cells	UCB	Completed	Intracerebroventricular	45	South Korea
4	NCT01297218 [82]	Cells	UCB	Completed	Intracerebral	9	South Korea
5	NCT02833792 [83]	Cells	Bone marrow	Recruiting	Intravenous	40	USA
6	NCT04040348 [84]	Cells	Umbilical cord	Active, not recruiting	Intravenous	6	USA
7	NCT04482413 [85]	Cells	Adipose tissue	Not yet recruiting	Intravenous	80	USA
8	NCT04954534 [86]	Cells	UCB	Not yet recruiting	Intracerebroventricular	9	South Korea
9	NCT02672306 [87]	Cells	Umbilical cord	Unknown	Intravenous	16	China
10	NCT01547689 [88]	Cells	UCB	Unknown	Intravenous	30	China
11	NCT01696591 [89]	Cells	UCB	Unknown	Intracerebroventricular	9	South Korea
12	NCT04228666 [90]	Cells	Adipose tissue	Withdrawn due to COVID-19 pandemic	Intravenous	24	USA
13	NCT04855955 [91]	Cells	Adipose tissue	Completed	N/A	1	USA
14	NCT04388982 [92]	Cells	Adipose tissue	Recruiting	Nasal drip	9	China
15	NCT02899091 [93]	Cells	N/A	Recruiting	Intravenous	24	South Korea
16	NCT04684602 [94]	Cells	N/A	Recruiting	N/A	5000	USA

## Data Availability

Not applicable.
